# Synthesis, Crystal Structure and DFT Studies of a New Dinuclear Ag(I)-Malonamide Complex

**DOI:** 10.3390/molecules23040888

**Published:** 2018-04-11

**Authors:** Saied M. Soliman, Assem Barakat, Mohammad Shahidul Islam, Hazem A. Ghabbour

**Affiliations:** 1 Department of Chemistry, Rabigh College of Science and Art, King Abdulaziz University, P. O. Box 344, Rabigh 21911, Saudi Arabia; 2 Department of Chemistry, Faculty of Science, Alexandria University, P. O. Box 426, Ibrahimia 21321, Alexandria, Egypt; 3 Department of Chemistry, College of Science, King Saud University, P. O. Box 2455, Riyadh-11451, Saudi Arabia; shahid10.amu@gmail.com; 4 Department of Pharmaceutical Chemistry, College of Pharmacy, King Saud University, P. O. Box 2457, Riyadh 11451, Saudi Arabia; ghabourh@yahoo.com; 5 Department of Medicinal Chemistry, Faculty of Pharmacy, Mansoura University, Mansoura 35516, Egypt

**Keywords:** silver (I) complex, malonamide, AIM, NBO, continuous shape measure

## Abstract

The synthesis and structural aspects of a new dinuclear silver (I) complex with malonamide type ligand (**L**) is reported. Each Ag ion in the [**Ag_2_L_2_(NO_3_)_2_]·H_2_O** complex is coordinated to two ligands, **L**, each acting as a bridged ligand via its two pyridine arms; Ag(I) acts as a connector between them. Two types of Ag-ligands close contacts were detected: Ag–N1, Ag–N4 from the two **L** units, and Ag–O5, Ag—O6 from the two nitrate anions, wherein both the nitrate ions are inside the cage formed by the **[Ag_2_L_2_]** unit. The coordination geometry around each Ag(I) is a distorted tetrahedron. The **[Ag_2_L_2_(NO_3_)_2_**] complex units are connected by weak intermolecular C—H…O interactions. The different intermolecular interactions were quantified using Hirshfeld surface analysis. Using two DFT methods (B3LYP and WB97XD), the nature and strength of the Ag–N and Ag–O interactions were described using atoms in molecules (AIM) and natural bond orbital (NBO) analyses. Topological parameters indicated that the strength of the two Ag–N bonds was similar, while that of the two Ag–O interactions were significantly different. Moreover, the Ag–N interactions have a predominant covalent character, while the Ag–O interactions are mainly ionic. The NBO analysis indicated that the most important anti-bonding Ag-orbital in these interactions has an s-orbital character.

## 1. Introduction

*N*,*N*-Malonamide derivatives (MAD) are important scaffolds present in many pharmaceuticals, synthetic and natural products. MAD have possessed several of the biological targets including selective and effective targeting to the γ optical receptor [1] and has been proved to be effective in treating Alzheimer’s disease [2] and cancer [3]. In addition, these compounds were identified as potent inhibitors of the α-glucosidase enzyme [4,5]. On the other hand, they find notable applications in peptidomimetic compound synthesis [6,7,8]. These compounds have the ability to bind with alkali, alkaline earth and transition metals, thus representing excellent ionophores for the construction of alkaline earth and alkali cation-selective electrodes [9]. Additionally, malonamide motifs have been used for the “green” extraction of some heavy metals [10].

**Scheme 1:**
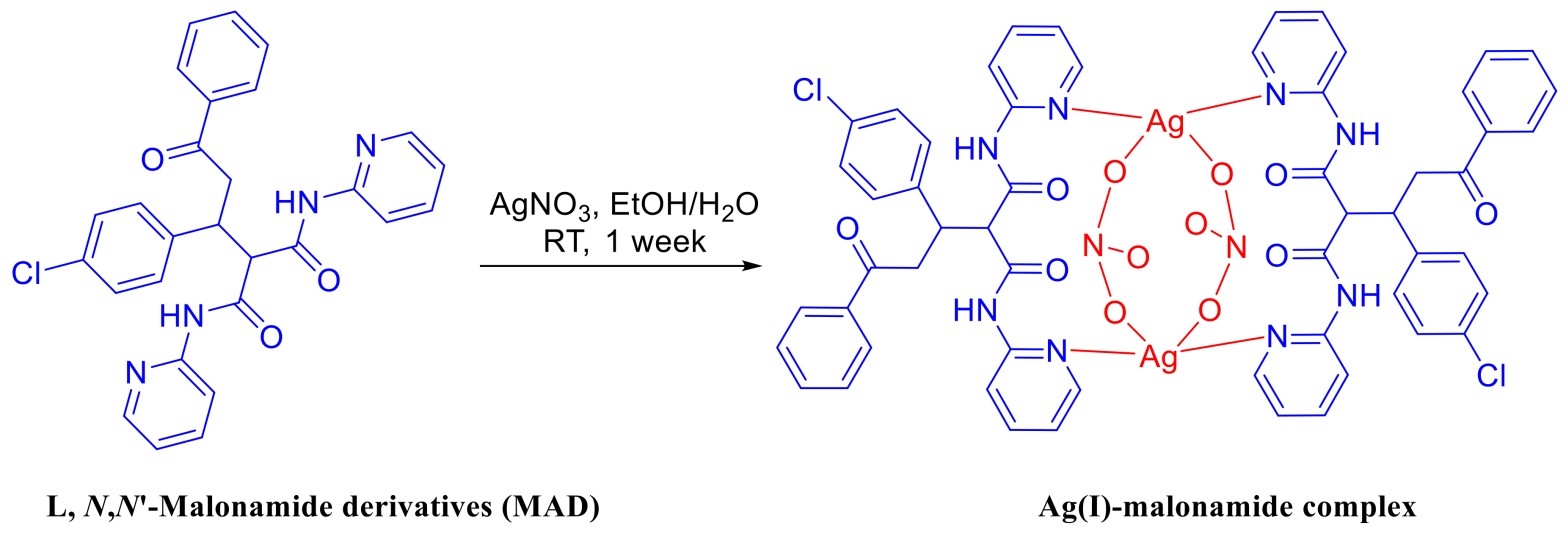
Schematic representation of the ligand (**L**) and its **[AgL(NO_3_)]_2_·H_2_O** complex.

On other hand, there is increasing interest in the biological activity of silver and its compounds as potent antibacterial and antifungal agents [11,12,13,14,15,16,17,18,19,20,21,22,23,24,25,26]. The structural chemistry of silver (I) complexes is also interesting, as Ag(I) could adopt variable coordination numbers (*n* = 2–6). The tetra-coordinated Ag(I) complexes are present in either tetrahedral or square planar coordination environment, the latter being less common. In addition, Ag(I) could form complexes with higher coordination numbers like octahedral or an trigonal prism or an intermediate structure between these two extremes [27]. To the best of our knowledge, there are no reported silver(I) complexes with such malonamide type ligand and, in the light of the increasing interest with silver (I) compounds, this work aims to synthesize a new Ag(I) complex with MAD ligand (**L**, Scheme 1). The new complex is characterized using FTIR, NMR and single crystal X-ray analyses, combined with DFT calculations. In addition, the nature and strength of the Ag-ligand interactions are discussed using natural bond orbital (NBO) and atoms in molecules (AIM) analyses.

## 2. Results

### Preparation of the Ligand (L) and Ag Complex

The ligand **L** [*N*,*N*-Malonamide derivatives (MAD)] is prepared following the method, previously reported by Barakat et. al. [5]. Mixing of a solution of **L** in ethanol, to a solution of AgNO_3_ in distilled water, the Ag-complex was collected from the solution after one week, as a colorless crystals of **[Ag_2_L_2_(NO_3_)_2_**]**·H_2_O** complex (Scheme 1). The target Ag-complex is photochemical and air stable.

## 3. Discussion

### 3.1. X-ray Crystal Structure

**Figure 1:**
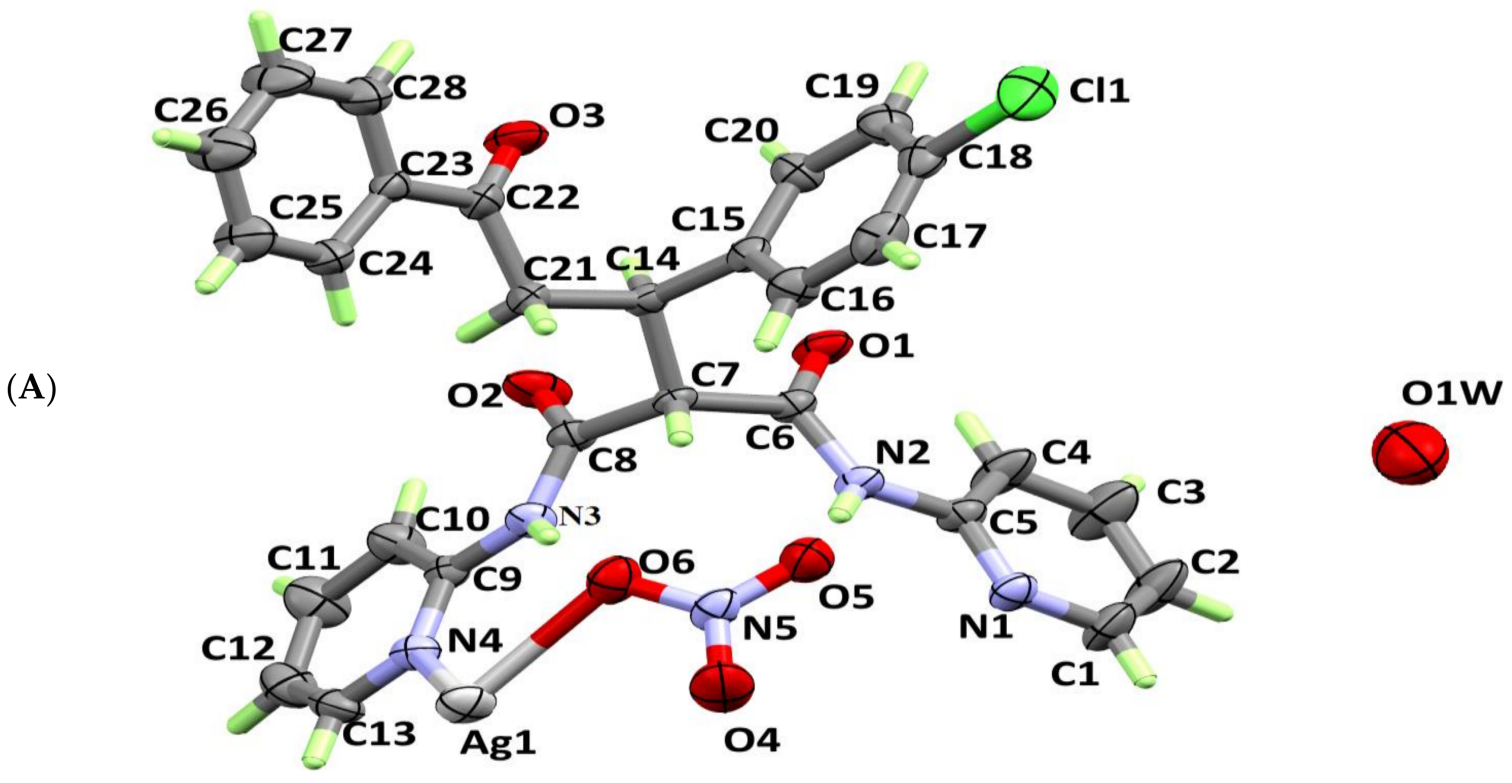

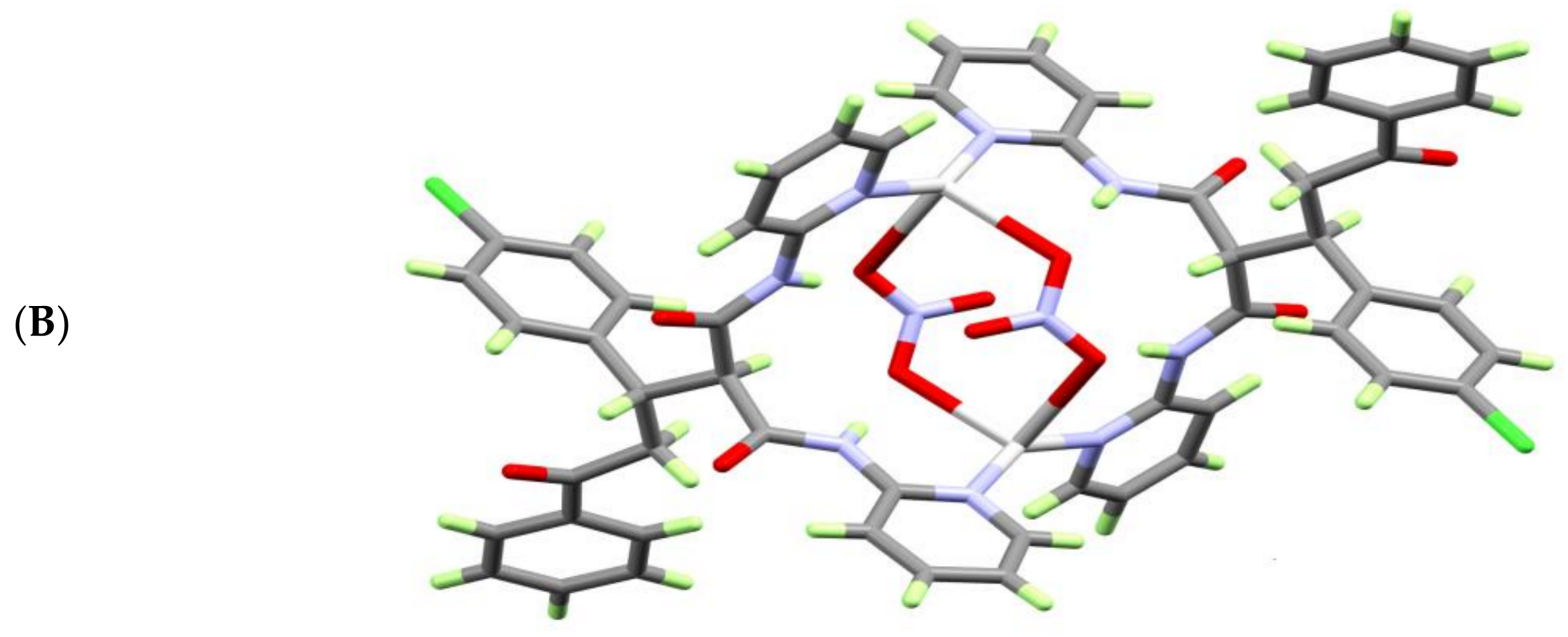
The atom numbering scheme of the asymmetric unit (**A**) and the coordination environment around the Ag(I) ion of **[AgL(NO_3_)]_2_·H_2_O** complex (**B**).

**Figure 2:**
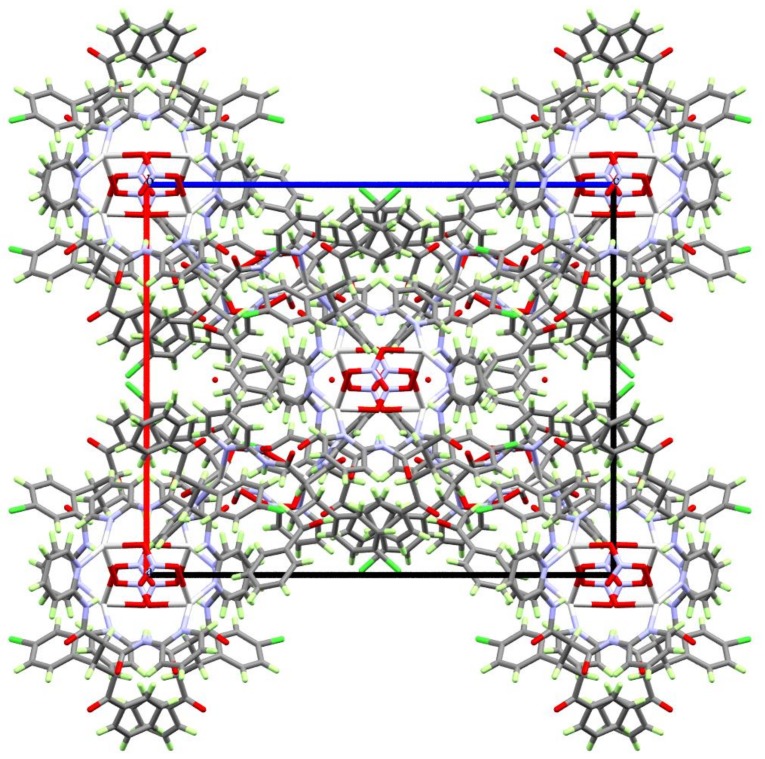
Packing of the complex units along the crystallographic *b*-direction showing the nitrate ions within the channels made by the **[AgL]_2_** complex units.

X-ray crystallographic analysis showed that the studied complex crystallizes in the tetragonal crystal system and I4_1_/a space group. The crystal parameters and structure refinement parameters are summarized in Table 1. Selected interatomic distances and angles are listed in Table 2. The asymmetric unit of the studied complex contains one **[AgL(NO_3_)]** unit and one half (the oxygen atom lies on a two-fold axis) of a disordered water molecule with un-refinable protons (Figure 1A). Each Ag is coordinated to two **L** units acting as a bridged ligand through the nitrogen atoms (N1 and N4) from the two pyridine rings, with the Ag(I) acting as a connector between them (Figure 1B). Two silver (I)-ligands interactions were observed in the crystal: Ag–O5 (2.720 Å) and Ag—O6 (2.541 Å) from two nitrate ions as well as two nearly identical Ag–N1 (2.221 Å) and Ag—N4 (2.224 Å) bonds from two ligand units. As a result, the Ag(I) is tetra-coordinated, with two N-atoms from two **L** units and two O-atoms from two nitrate groups, leading to a more likely distorted tetrahedral coordination geometry. Interestingly, the **[Ag_2_L_2_(NO_3_)_2_**] complex showed a huge cyclic structure formed by the **L—Ag—L—A**g moieties, with two nitrate ions locating inside it and connecting the two silver ions. This leads to the interesting feature of packing shown in Figure 2, where the nitrate ions are located inside the channels made by the **[Ag_2_L_2_**] complex cation.

**Figure 3:**
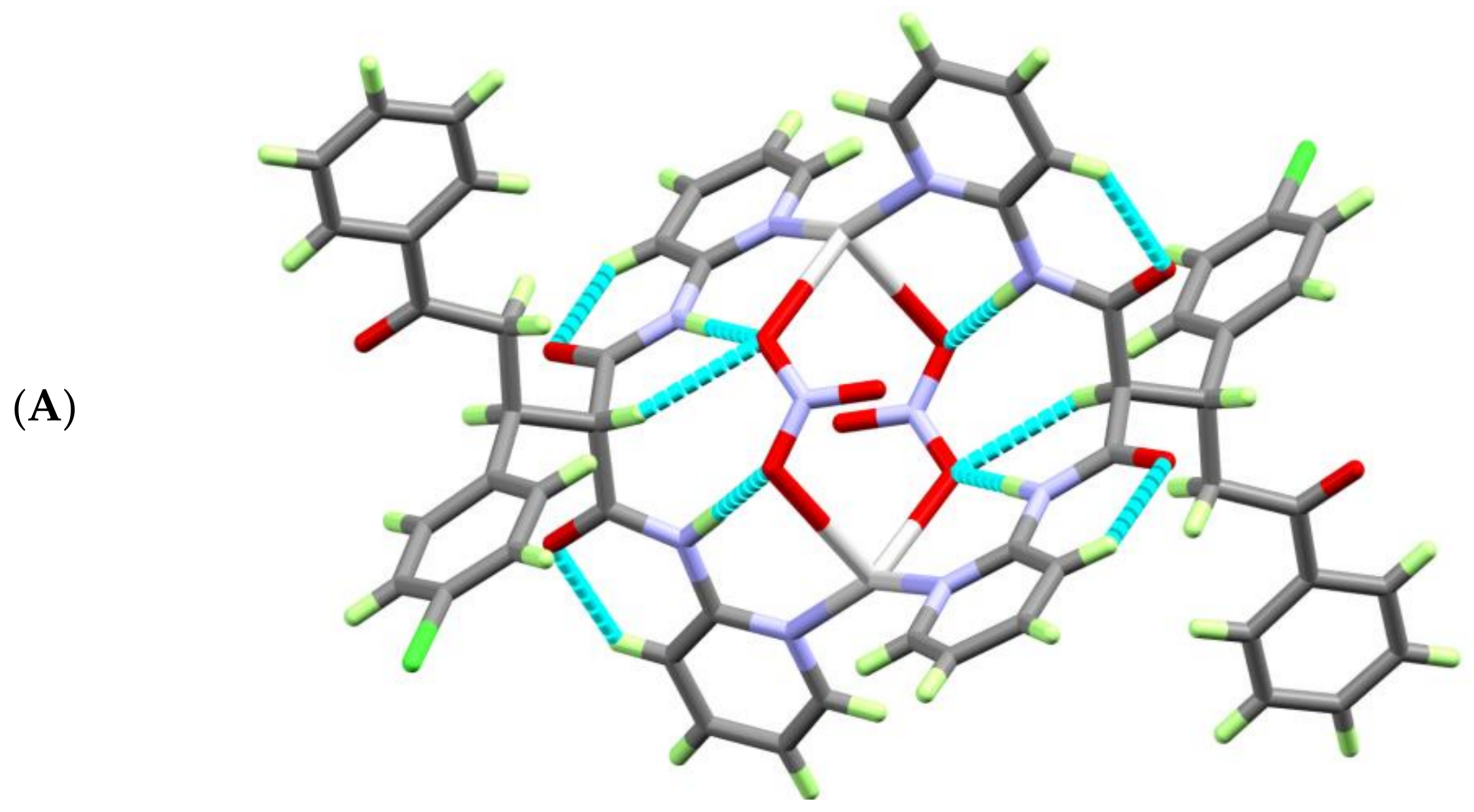

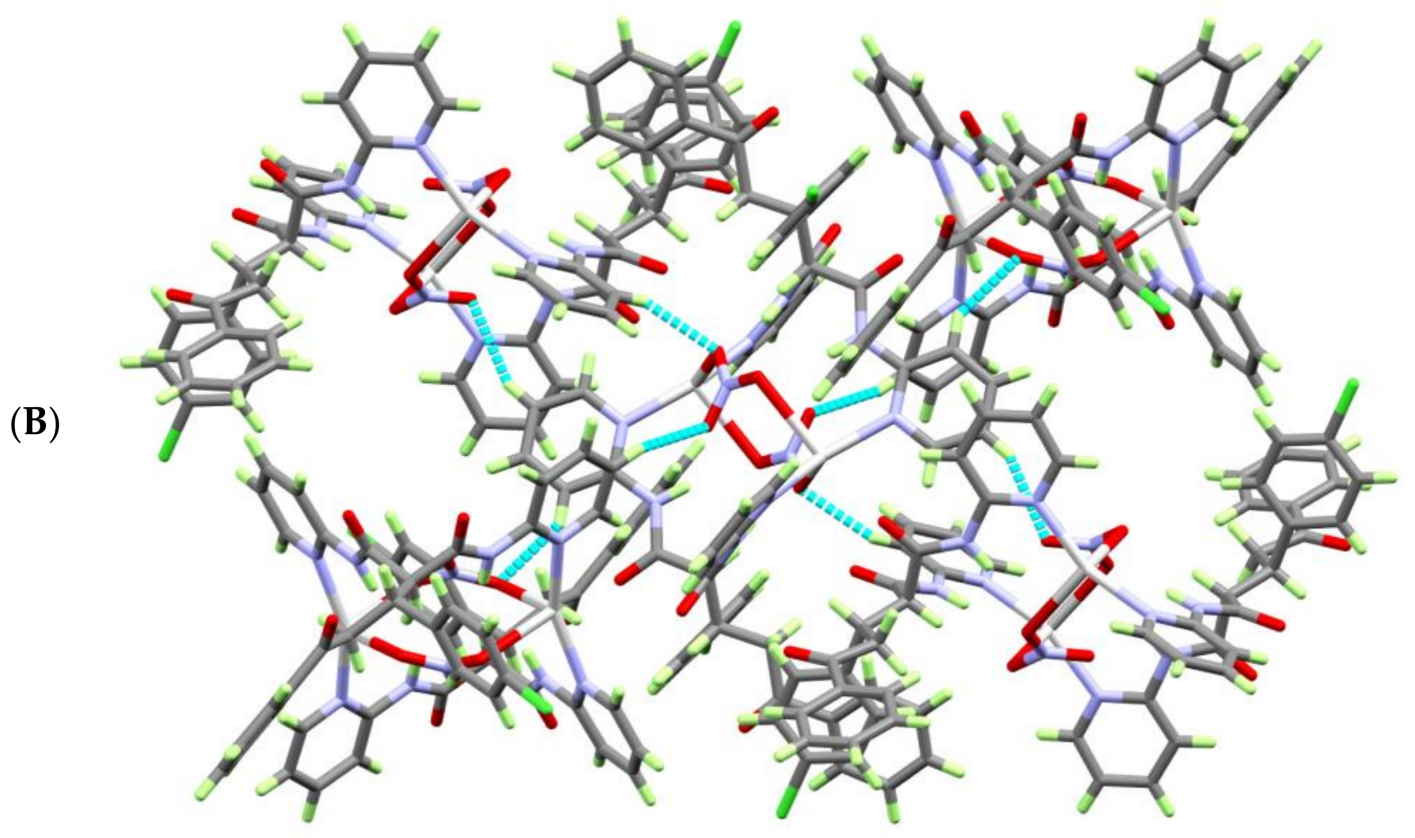
View of the molecular packing of the **[AgL(NO_3_)]_2_·H_2_O** complex units showing: (**A**) the intramolecular C—H…O and N—H…O and (**B**) intermolecular C4—H4A…O1 and the C4—H4A…O6ii H-bonding interactions.

The structure of **[Ag_2_L_2_(NO_3_)_2_**] unit is stabilized by a set of intramolecular C—H…O and N—H…O hydrogen bonding interactions (Figure 3A) formed between the NH proton from the ligand units and the O-atoms from the nitrate anions (Table 3). The molecules are packed by the two non-classical C2—H2A…O4 and C4—H4A…O6 intermolecular hydrogen bonding interactions shown in Figure 3B and listed in Table 3.

### 3.2. Hirshfeld (HF) Analysis of Molecular Packing

**Figure 4:**
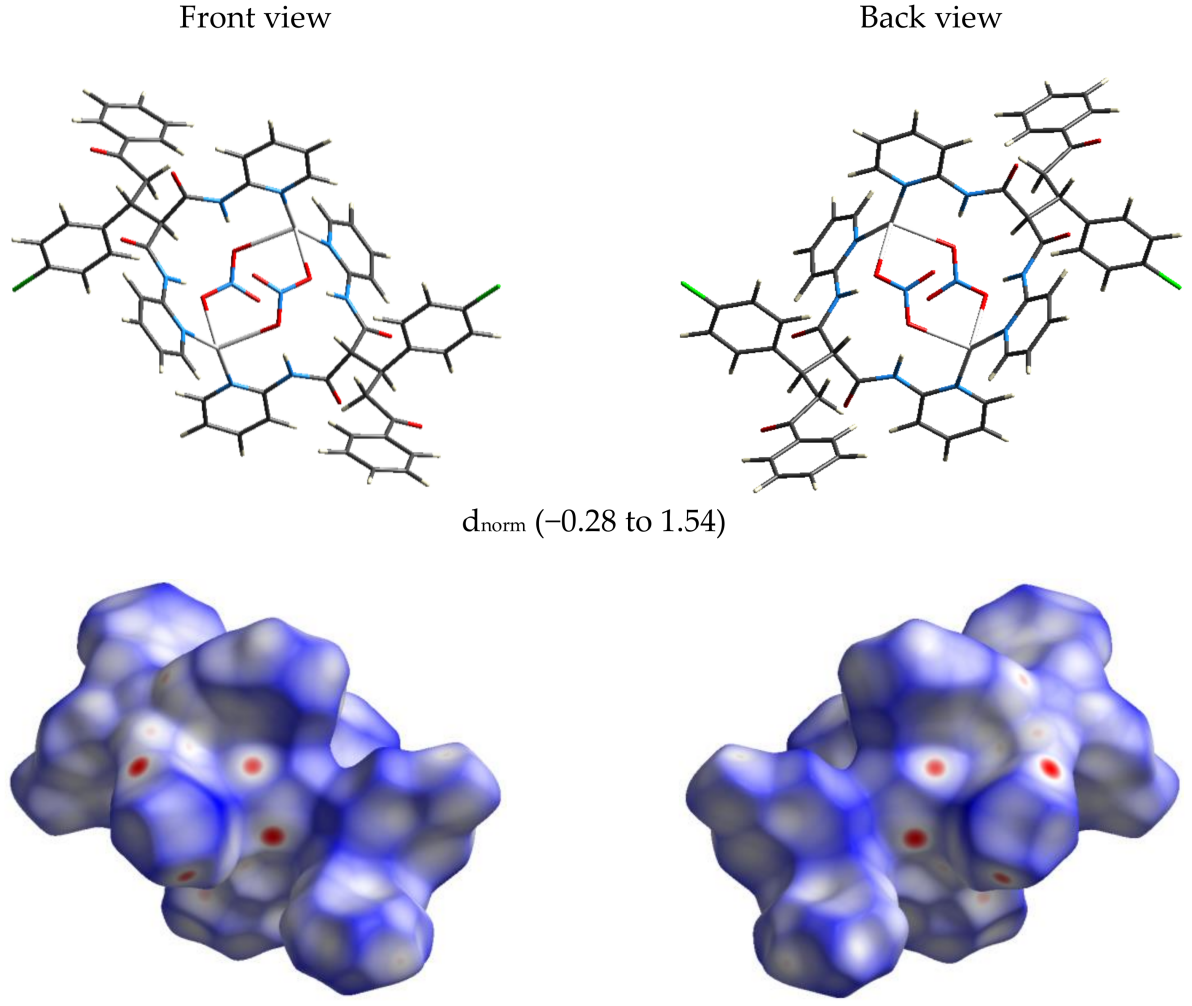
The d_norm_ Hirshfeld surface of **[AgL(NO_3_)]_2_.H_2_O** complex.

**Figure 5:**
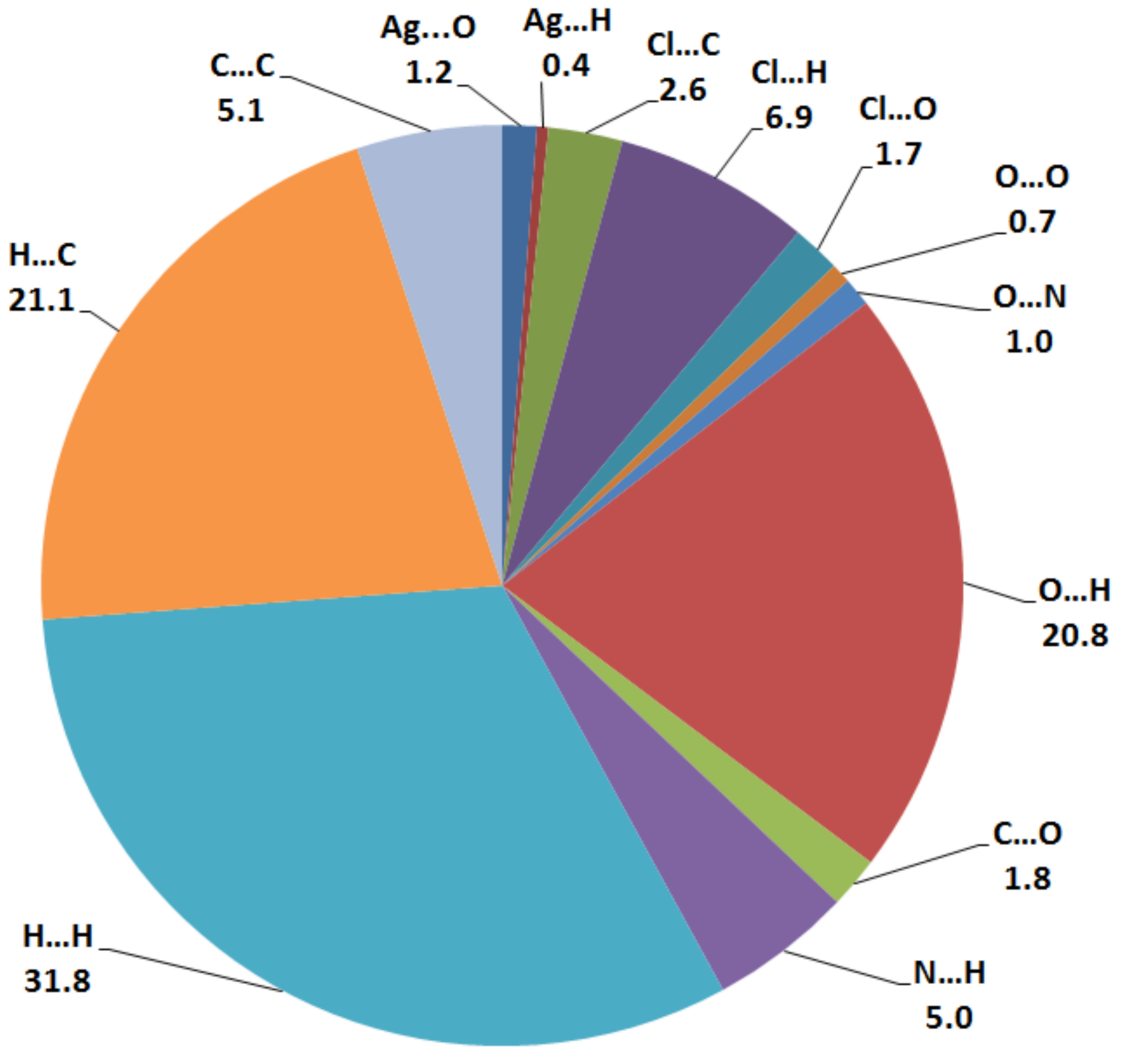
Percentage of possible contacts in the **[AgL(NO_3_)]_2_·H_2_O** complex.

**Figure 6:**
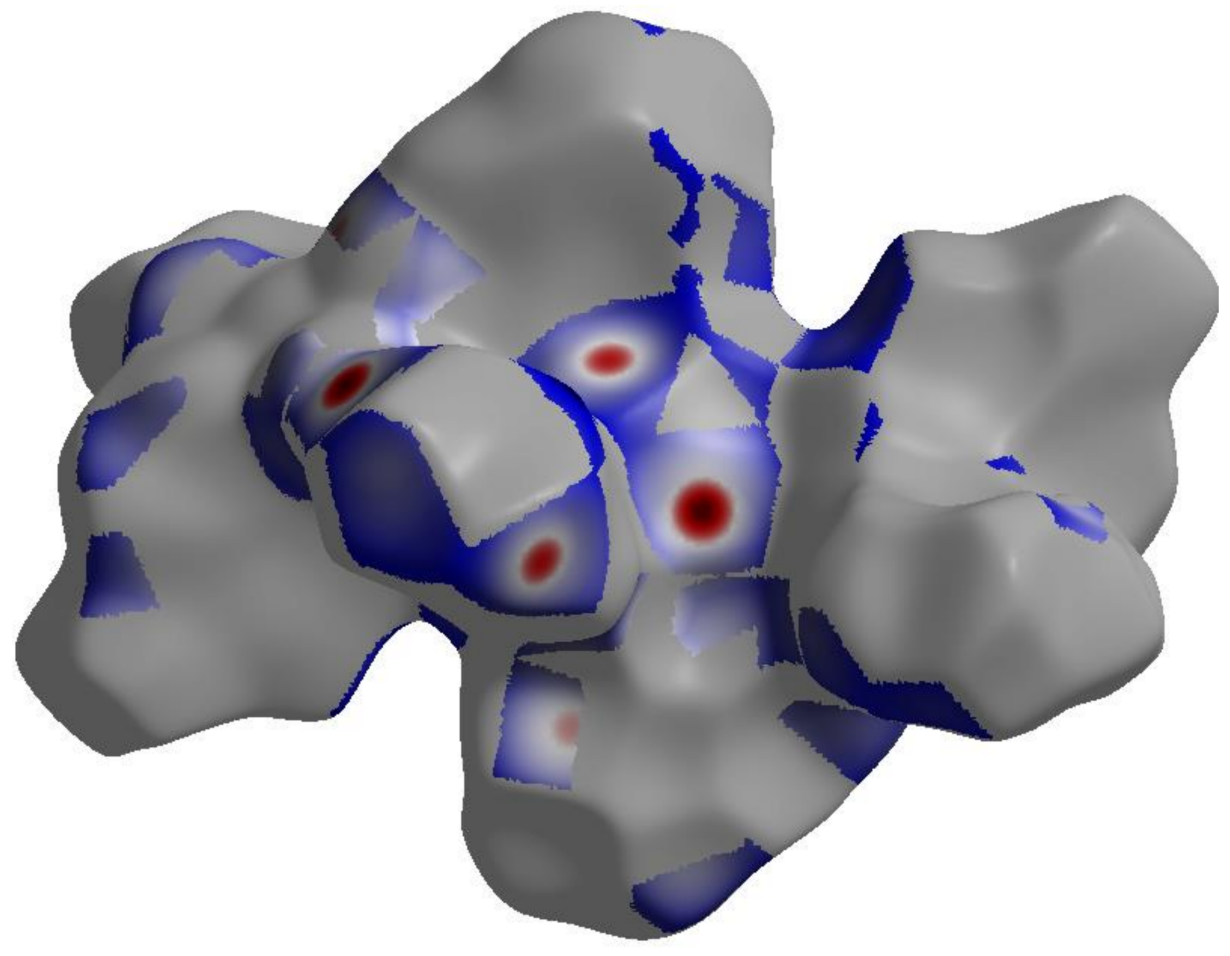
The significant O…H interactions (dark red regions) in the decomposed d_norm_ map.

The nature and amount of intermolecular interactions in **[Ag_2_L_2_(NO_3_)_2_**] crystal structure were described using Hirshfeld surface analysis (Figure 4 and Figure S1). The d_norm_, shape index (SI) and curvedness maps [28,29,30,31] were used to describe the importance of non-covalent interactions in the molecular packing of **[Ag_2_L_2_(NO_3_)_2_**] complex. With the aid of the full and decomposed fingerprint plots shown in Figure S2, the percentage values calculated for all possible intermolecular contacts observed in the crystal structure of **[Ag_2_L_2_(NO_3_)_2_**] complex are shown in Figure 5. According to the shape index and curvedness maps shown in Figure S1, the π–π stacking interactions were almost neglected. The amount of C…C contacts were 5.1%, which appeared as blue regions in the decomposed d_norm_ map indicating that this interaction is weak (Figure S3). The major interactions occurring in the crystal were found to be the H…H, C…H and O…H contacts, which have 31.8, 21.1 and 20.8%, respectively, from the overall fingerprint plot. The H…H interactions were proved to play an important role in the crystal stability [32]. The O…H interactions appeared in the d_norm_ map as strong red spots (Figure 6), indicating their significance in the molecular packing, while the C…H interactions appeared to be weak (Figure S3). The O…H contact distances are 2.355 Å and 2.234 Å for the O4…H2A and O6…H4A interactions, respectively. Another two polar interactions contributed by 6.9% and 5.0% from the overall fingerprint, due to the Cl…H and N…H intermolecular interactions, respectively. The former appeared as fade red spots, while the latter appeared as blue colored regions, indicating that these interactions were weak and insignificant, respectively (Figure S4). The rest of the intermolecular contacts made a small contribution to the overall fingerprint plot and appeared insignificant.

### 3.3. FTIR and NMR Spectra

The FTIR spectra of the compounds, recorded as KBr pellets, are shown in Figure S5. The water of crystallization appeared as a broad band at 3445 cm^−1^ in accordance with the reported X-ray structure. The infrared spectra of the free ligand showed ν_(NH)_ at 3309 and 3267 cm^−1^. In the case of the complex, a signal due to the N—H stretching vibration was detected at 3277 cm^−1^. The shift of the NH stretching frequency in the complex could be attributed to its intramolecular H-bonding with NO_3_¯ counter anions. The infrared spectra of the ligand **L** display a band at 1678 cm^−1^ due to the stretching vibration of the conjugated C=O groups. Interestingly, in the complex, we noted two C=O stretching vibrations at higher wavenumbers of 1712 and 1693 cm^−1^. The band at 1712 cm^−1^, possibly due to the malonamide motif, does not correspond to a conjugated C=O stretching vibration. Hence, the band at 1693 cm^−1^ could be attributed to the stretching vibration of the conjugated C=O of the benzoyl moiety. In the silver complex, the new band at 1383 cm^−1^ is attributed to the N—O stretching vibration of the nitrate group.

The NMR spectra of the malonamide complex are provided in Figure S6. The assignment of the ^1^H-NMR spectra of the free and coordinated ligand is tabulated in Table 4, which showed that the NMR chemical shifts are similar in both compounds as the complexation between Ag(I) with O and N donor ligands are relatively weak, and it did not affect the NMR spectra of the ligands Table 4.

### 3.4. AIM Analyses

AIM is the most suited method to illustrate the strength and nature of interactions between atoms in molecular systems. The strength and nature of the Ag—N(**L**) and Ag—O(nitrate) interactions were described using the AIM topological parameters computed using B3LYP and WB97XD methods. The results of the topological parameters at the Ag–N and Ag–O bond critical points are summarized in Table 5. Due to symmetry consideration, only half of the Ag–N and Ag–O bonds were discussed. The compound has two Ag–N interactions differing very slightly in the Ag–N distances. The total electron density (ρ(r)) values at the Ag–N BCPs are almost equivalent and the results of the topological parameters for both the bonds are similar. As a result, the strength of the Ag–N bonds in the complex was almost equivalent [33]. In contrast, the Ag(I) complex showed two significantly different Ag–O interactions. At the corresponding BCPs, the short Ag—O6 (2.539 Å) had total electron density (ρ(r)) of 0.0315–0.0318 a.u., compared to the longer Ag—O5 (2.720 Å), from the same nitrate ion, with ρ(r) of 0.0220–0.0222 a.u. The shorter Ag–O6 interaction had higher total electron density (ρ(r)) and higher interaction energy (E_int_) than the longer one. The interaction energies at the BCPs were estimated using the relationship, E_int_ = V(r)/2 [34]. These results indicated that the nitrate anion acts as a bridged ligand connecting the two silver atoms, with one weak and one strong Ag–O interaction. The WB97XD method contains correction for the dispersion interactions, which is not the case for the B3LYP one. Since the topological analysis results were similar for both the methods, the AIM topological parameters were nearly independent of the dispersion effects of the studied system.

The ratio |V(r)|/G(r) and the total energy density H(r) are useful parameters for bond nature description. The total energy density H(r) is negative for covalent bonding, while positive values indicate closed-shell interactions. In addition, the ratio |V(r)|/G(r) is more than 2 for typically covalent interactions [35]. In general, |V(r)|/G(r) > 1 indicates dominant covalent bonding and |V(r)|/G(r) < 1 indicates predominant closed-shell interactions. On the basis of H(r) and |V(r)|/G(r), all of the Ag–N bonds could be described as predominantly covalent. Interestingly, the slightly shorter Ag–N bond has slightly more negative H(r) value and also higher |V(r)|/G(r) ratio. In agreement with the fact that the shorter the atom–atom distance, the greater the orbital overlap between the atoms, the more bond covalency could be predicted. In contrast, the Ag–O interactions showed positive H(r) and |V(r)|/G(r) < 1, indicating the ionic character of these interactions.

### 3.5. Natural Bond Orbital (NBO) Analyses

Natural bond orbital calculations on the X-ray structure of the Ag(I) complex yielded information on the natural atomic charges and the most important intramolecular donor (**L**/NO_3_^−^)-acceptor(**Ag**) charge transfer (ICT) interactions. The NBO method is the best suited for atomic charge calculations, as it is less sensitive to basis set variations. Table S1 shows the natural charges obtained using the B3LYP and WB97XD methods. The net natural charges at the silver ion and ligand (**L**) unit as well as the anion (NO_3_¯) were in the range, 0.5966–0.6181 e, 0.2173–0.2040 e and −0.8139 to −0.8221 e, respectively. These values indicated that the silver(I) charge was significantly reduced by the electron density transferred from the ligand/anion groups. The amount of charge transferred from the ligand/anion to the silver(I) was in the range, 0.3819–0.4034 e. The amount of electron density transferred from the ligand (**L**) is higher (0.2173–0.2040 e) than that transferred from the nitrate anion (0.1779–0.1861 e).

Other features related to the strength and nature of the Ag–N and Ag–O interactions could be obtained from the second order perturbation results. The stabilization energies (E^(2)^) resulting from the donor (NBO_i_)-acceptor (NBO_j_) charge transfer interactions involved in the Ag–N and Ag–O interactions are presented in Table 6. Charge transfer interactions with higher E^(2)^ values were stronger than those with lower E^(2)^ values [36]. From Table 6, the two LP(1)N→LP *Ag interactions of the two ligand (L) molecules were found to have similar interaction energies. The net value of these Ag–N interactions were very high indicating strong interactions between the LP(1)N NBO with the silver anti-bonding NBOs (LP*), whereas the shorter Ag–N bond had slightly higher interaction energy than the longer one. On other hand, each nitrate showed two different Ag–O bonding interactions. The short Ag–O interaction had higher E^(2)^ value (32.98 kcal/mol) than the longer one (20.11 kcal/mol). In both the cases, the donor–acceptor interactions from the O-atom second lone pair (LP(2)) are higher than those from its first lone pair (LP(1)) orbital. Comparing the B3LYP and WB97XD methods, the latter was found to overestimate the interaction energies. ICT from the metal filled orbitals to the ligand antibonding ones were not observed. As a result, the Ag(I)→L π-back donation interaction were negligible.

**Figure 7:**
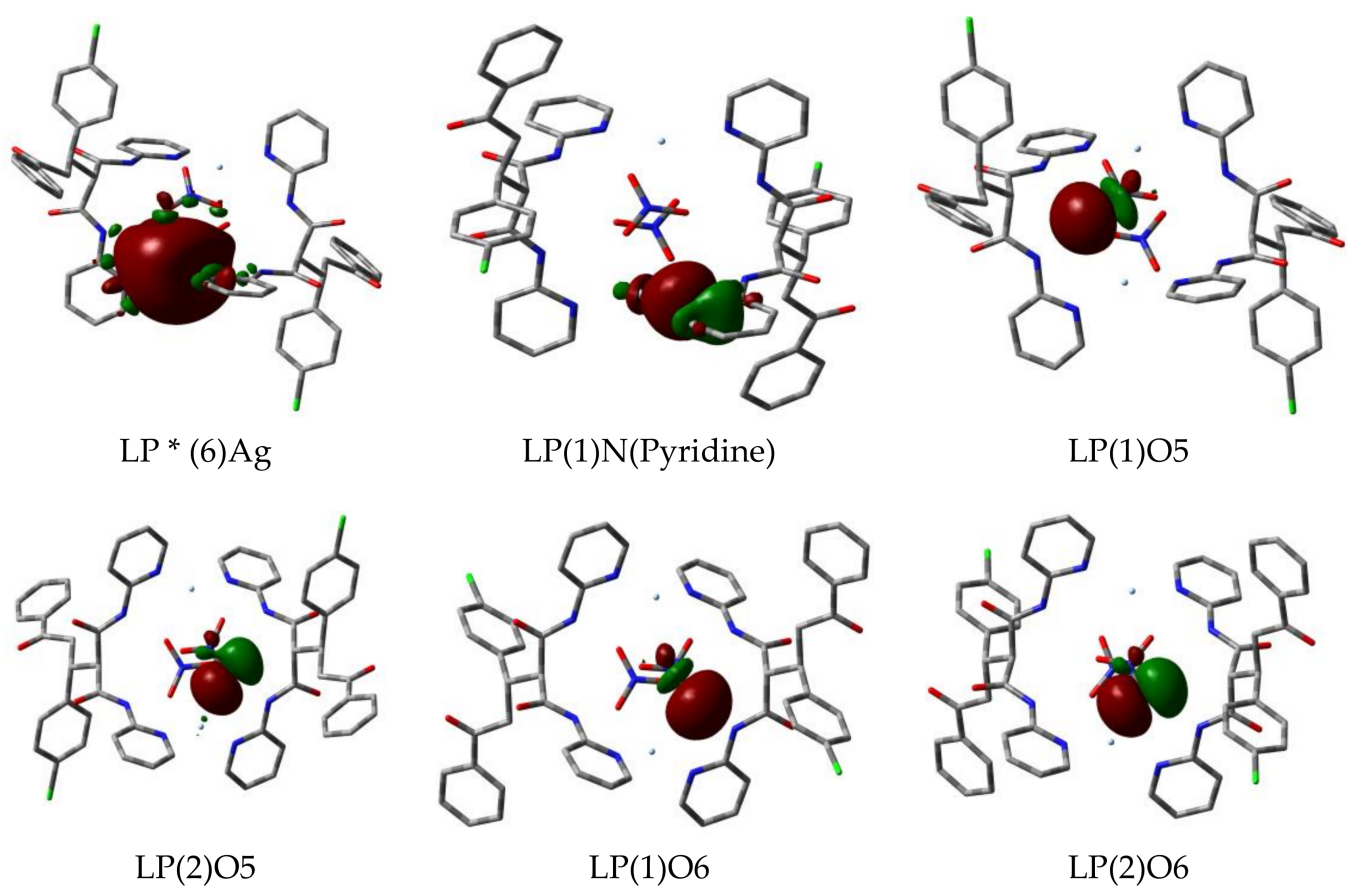
The most important natural orbitals included in the Ag–N and Ag–O interactions of the **[AgL(NO_3_)]_2_** complex. The near-spherical shape of the LP * (6)Ag anti-bonding natural orbital density can be noted.

On other hand, due to these donor (NBO_i_)-acceptor (NBO_j_) interactions between the donor atoms from the ligand groups and the Ag(I) ion, the occupancy and energy of the NBOs involved in these interactions showed significant variations. Table 7 showed the occupancy and energy of the different natural orbitals (NBOs) included in the Ag–N and Ag–O interactions compared to those in the corresponding free **L**, nitrate or Ag(I) ions. The occupancy of all the donor atom NBOs were found to be lowered while that of the anti-bonding NBOs (LP * Ag) increased. The Ag(I) anti-bonding NBO affected the most by the interactions with the donor atoms nonbonding orbitals is LP * (6). In addition, the energies of all ligand-filled nonbonding orbitals and most of the Ag(I) anti-bonding orbitals are stabilized compared to the free species. Figure 7 shows a pictorial representation of the nonbonding orbitals from the ligand donor atoms and the anti-bonding orbital (LP * (6)) from Ag(I) with a spherical orbital density due to its s-orbital character.

### 3.6. Continuous Shape Measure (CShM)

Silver (I) coordination geometries varied significantly depending on the nature of the ligand and counter anion. The coordination geometry of the tetra-coordinated silver (I) ion could be either tetrahedral or square planar. The latter geometry is rarely reported. In both the cases, deviation is possible. Here, we used the criteria of the continuous shape measurements (CShM) [37] to assign the coordination geometry around Ag(I) in the studied complex. The values of shape measurements of the coordination environment around Ag(I) were 3.168 and 24.331, with respect to the ideal tetrahedron and square planar, respectively. Larger value of shape measurement (24.331) with respect to ideal square planar geometry was obtained than that for the ideal tetrahedral (3.168). As a result, the coordination environment around Ag(I) is more likely to be distorted tetrahedron than square planar.

## 4. Materials and Methods

### 4.1. Materials

Silver(I) nitrate was purchased from Sigma-Aldrich (Riedstraße, Germany). Infrared spectra (4000–400 cm^−1^) were recorded on an Alpha Bruker instrument in KBr pellets and the wavenumbers are in cm^−1^. Perkin Elmer 2400 Elemental Analyzer (PerkinElmer, Inc., 940 Winter Street, Waltham, MA, USA) was used for CHN elemental analysis. JEOL-400 NMR spectrometer (Japan, Tokyo) was used for recording NMR spectra in DMSO-*d*_6_. Detailed analytical results are given in the supplemental data.

### 4.2. Synthesis of the Silver(I) Complex

To a solution of **L** (Malonamide derivative) (~0.499 g, 1 mmol) in 10 mL ethanol, solution of AgNO_3_ (~0.170 g, 1 mmol) in 5 mL distilled water was added. After one week, (air and light stable) colorless crystals of **[Ag_2_L_2_(NO_3_)_2_**]**.H_2_O** complex were collected from the solution.

Yield: 80%; IR (ν_max_, cm^−1^): **[AgL(NO_3_)]_2_.H_2_O:** 3445, 3277, 1712, 1693, 1384; Anal. Calcd. C_56_H_48_Ag_2_Cl_2_N_10_O_13_: C, 49.61; H, 3.57; N, 10.33%. Found: C, 49.60; H, 3.58; N, 10.32%. ^1^H-NMR for [AgL(NO_3_)]_2_.H_2_O (400 MHz, DMSO-*d*_6_) *δ*: 3.26 (d, 1H, *J* = 16.1 Hz, CH), 3.71–3.78 (dd, 1H, *J* = 16.1 Hz, 9.52 Hz, CH), 4.18(m, 2H, CH_2_), 7.06 (t, 1 s, *J* = 5.9 Hz, Ar—H), 7.15 (t, 1H, *J* = 6.2 Hz, Ar—H), 7.23 (d, 1H, *J* = 4.8 Hz, Ar—H), 7.34 (d, 1H, *J* = 8.1 Hz, Ar—H), 7.43 (t, 1H, *J* = 7.3 Hz, Ar—H), 7.56 (t, 1H, *J* = 6.6 Hz, Ar—H), 7.68 (t, 1H, *J* = 8.8 Hz, Ar—H), 7.75 (d, 1H, *J* = 8.8 Hz, Ar—H), 7.83(d, 1H, *J* = 8.1 Hz, Ar—H), 8.04 (d, 1H, *J* = 8.0 Hz, Ar—H), 8.24 (d, 1H, *J* = 4.4 Hz, Ar—H), 8.33 (d, 1H, *J* = 5.1 Hz, Ar—H), 10.48 (s, 1H, NH), 10.62 (s, 1H, NH).

### 4.3. Computational Methods

Using the X-ray geometry, the B3LYP [38] and WB97XD [39] were employed to investigate the strength and nature of the Ag–N and Ag–O interactions in the target complex. Gaussian built in split valence triple-zeta 6-311G(d,p) basis sets [40] were used for nonmetal atoms, while the Hay–Wadt relativistic effective core potentials LANL08 [41] were used for Ag. The latter basis set was obtained from EMSL basis set exchange [42]. All calculations were performed using a Gaussian 09 program [43,44] imposing C_i_ symmetry. Topology analysis using the AIM [45] method was performed with the aid of the Multwfn program [46]. The NBO calculations were made using the NBO 3.1 [47] embedded in Gaussian 09.

### 4.4. X-ray Measurements

A Bruker D8 Venture area diffractometer (Bruker AXS GmbH, Karlsruhe, Germany) with graphite monochromated Mo-K*α* radiation, λ = 0.71073 Å at 100 (2) K is used for data collection. Data reduction and cell refinement were carried out using Bruker SAINT. The crystal structure of the silver (I) complex is solved by SHELXS-97 [48,49]. The CIF file CCDC-1543617 contains the crystallographic data for this Ag(I)-complex.

### 4.5. Hirshfeld Surface Analysis

Hirshfeld surfaces and 2D fingerprint plots were analyzed using Crystal Explorer 3.1 program [50], in order to quantify the different intermolecular interactions present in **[Ag_2_L_2_(NO_3_)_2_**] complex crystal. The d_norm_, shape index and curvedness maps, as well as the fingerprint plots were drawn using the same software.

## 5. Conclusions

A new Ag(I) MAD complex (**[Ag_2_L_2_(NO_3_)_2_**]**·H_2_O**) has been synthesized for the first time and characterized spectroscopically. The chemical structure of the synthesized complex, deduced by a single crystal X-ray diffraction technique, shows that the silver (I) ion is bound to two *N* and two *O* atoms. Structural studies in the framework of the AIM and NBO methods were performed to describe the nature and strength of the Ag–ligands interactions. Topological parameters obtained from both methods (B3LYP and WB97XD) show that each Ag-atom has two significantly different Ag–O interactions and almost two equivalent Ag–N bonds. The former are mainly ionic while the latter ones are predominantly covalent, based on AIM analysis. The NBO method shows that these interactions occur between the lone pair orbitals of the ligand donor atom and the LP * (6) anti-bonding orbital of the Ag-atom. The latter has mainly s-orbital character.

## Figures and Tables

**Table 1:** The crystal and experimental data of **[AgL(NO_3_)]_2_.H_2_O** complex.

Parameters	[AgL(NO_3_)]_2_.H_2_O]
F.wt.	C_56_H_48_Ag_2_Cl_2_N_10_O_13_
M.wt.	1355.68
T	293(2) K
λ (Mo *K*α radiation)	0.71073 Å
Crystal system	Tetragonal
Space group	I4_1_/a
Unit cell dimensions	*a* = 21.1544 (11) Å*c* = 25.2931 (9) Å
Volume	11318.9 (12) Å^3^
R-Factor	0.153
Z	8
Density (calculated)	1.589 mg m^−3^
Absorption coefficient	0.86 mm^−1^
*F*(000)	5472
Crystal size	0.29 × 0.20 × 0.16 mm
Theta range for data collection	2.3–21.5°
Completeness to θ = 21.5°	99.9
Goodness-of-fit on *F^2^*	1.06
Diffractometer	Bruker APEX-II D8 Venture diffractometer
Absorption correction	Multi-scan, SADABS
Limiting indices	−27 ≤ h ≤ 27, −27 ≤ k ≤ 27, −32 ≤ l ≤ 32
Reflections collected/unique	77356/3477 [R(int) = 0.153]
Refinement method	Full-matrix least-squares on F^2^
Data/restraints/parameters	6500/0/2375
Final R indices [I > 2σ(I)]	R1 = 0.070, wR2 = 0.151
R indices (all data)	R1 = 0.152, wR2 = 0.190
Largest diff. peak and hole (e A^−3^)	0.83 and −0.73
CCDC number	1543617

**Table 2:** Selected geometric parameters (Å, °) of **[AgL(NO_3_)]_2_·H_2_O** complex.

Ag1—O6	2.541 (5)	O6—N5	1.247 (6)
Ag1—N4	2.224 (4)	N1—C1	1.348 (9)
Ag1—N1i	2.221 (5)	N1—C5	1.342 (8)
Cl1—C18	1.755 (7)	N2—C5	1.404 (7)
O1—C6	1.209 (7)	N2—C6	1.363 (8)
O2—C8	1.219 (8)	N3—C8	1.337 (7)
O3—C22	1.203 (9)	N3—C9	1.390 (6)
O4—N5	1.218 (7)	N4—C9	1.340 (7)
O5—N5	1.236 (7)	N4—C13	1.333 (8)
O6—Ag1—N4	101.67 (16)	N1—C5—N2	115.3 (5)
O6—Ag1—N1i	102.63 (16)	N1—C5—C4	121.9 (6)
N1i—Ag1—N4	152.93 (18)	O1—C6—C7	122.0 (5)
Ag1—O6—N5	115.2 (3)	N2—C6—C7	114.6 (5)
C1—N1—C5	117.3 (5)	O1—C6—N2	123.3 (5)
Ag1i—N1—C1	111.7 (4)	O2—C8—C7	120.5 (5)
Ag1i—N1—C5	130.9 (4)	N3—C8—C7	115.8 (5)
C5—N2—C6	127.5 (5)	O2—C8—N3	123.7 (5)
C8—N3—C9	128.1 (5)	N3—C9—N4	115.2 (5)
Ag1—N4—C9	127.3 (3)	N4—C9—C10	120.9 (5)
Ag1—N4—C13	114.3 (4)	N3—C9—C10	123.9 (5)
C9—N4—C13	118.2 (5)	N4—C13—C12	123.9 (7)
O4—N5—O5	122.1 (5)	Cl1—C18—C17	120.1 (5)
O4—N5—O6	118.4 (5)	Cl1—C18—C19	118.8 (6)
O5—N5—O6	119.4 (5)	O3—C22—C23	121.2 (6)
N1—C1—C2	123.7 (6)	O3—C22—C21	120.8 (6)
N2—C5—C4	122.7 (6)		

**Table 3:** Selected hydrogen-bonding parameters of **[AgL(NO_3_)]_2_.H_2_O** complex.

D—H···A	D—H (Å)	H…A (Å)	D…A (Å)	D—H…A (°)
N2—H2B…O5	0.8600	2.2300	3.044 (7)	158.00
N3—H3B…O6	0.8600	2.0800	2.923 (6)	167.00
C2—H2A…O4i	0.9300	2.4900	3.358 (11)	155.00
C4—H4A…O1	0.9300	2.2000	2.806 (8)	123.00
C4—H4A…O6ii	0.9300	2.3600	3.160 (8)	144.00
C7—H7A…O6	0.9800	2.4800	3.349 (7)	148.00

Symmetry code(s): (i) *y* + 5/4, −*x* + 1/4, *z* + 1/4; (ii) −*y* − 1/4, *x* − 3/4, *z* + 1/4.

**Table 4:** The full assignment of the ^1^H-NMR spectra of the free and coordinated ligand. Atom numbering refers to the X-ray structure.

Atom	L	[AgL(NO_3_)]_2_·H_2_O	Atom	L	[AgL(NO_3_)]_2_·H_2_O
H1A	8.34	8.33	H19A	7.57	7.56
H2A	7.14	7.15	H20A	7.44	7.43
H3A	7.86	7.83	H21A	3.24	3.26
H4A	8.24	8.24	H21B	3.66–3.70	3.71–3.78
H7A	4.15–4.28	4.18	H24A	8.08	8.04
H10A	8.24	8.24	H25A	7.68	7.68
H11A	7.86	7.83	H26A	7.80	7.75
H12A	7.14	7.15	H27A	7.68	7.68
H13A	8.34	8.33	H28A	8.08	8.04
H16A	7.44	7.43	HN2	10.56	10.48
H17A	7.57	7.56	HN3	10.65	10.62

**Table 5:** Topology parameters of the Ag–N and Ag–O interactions of **[AgL(NO_3_)]_2_·H_2_O** complex using two different DFT methods.

Bond	Distance Å	ρ(r) a.u.	G(r) a.u.	V(r) a.u.	H(r) a.u.	|V(r)|/G(r) ^a^	E_int_ Kcal/mol
**B3LYP**							
Ag—O6	2.541	0.0222	0.0210	−0.0199	0.0010	0.9514	6.2554
Ag—O5	2.720	0.0318	0.0346	−0.0337	0.0009	0.9748	10.5845
Ag—N1i	2.221	0.0708	0.0858	−0.0980	−0.0122	1.1419	30.7522
Ag—N4	2.224	0.0703	0.0854	−0.0974	−0.0119	1.1399	30.5452
**WB97XD**							
Ag—O6	2.539	0.0220	0.0211	−0.0201	0.0010	0.9529	6.2978
Ag—O5	2.720	0.0315	0.0348	−0.0339	0.0009	0.9746	10.6384
Ag—N1i	2.221	0.0706	0.0869	−0.0989	−0.0120	1.1385	31.0405
Ag—N4	2.222	0.0701	0.0864	−0.0982	−0.0118	1.1365	30.8184

^a^ |V(r)|/G(r): ratio of electron potential to kinetic energy density.

**Table 6:** The most significant donor (NBO_i_)-acceptor (NBO_j_) charge transfer interaction energies (kcal/mol) of the Ag–N and Ag–O interactions.

NBO_i_	NBO_j_	B3LYP	WB97XD
LP(1)N1^i^	LP * (6)Ag	33.56	39.38
LP(1)N1^i^	LP * (7)Ag	10.54	13.42
**Net**		**44.10**	**52.80**
LP(1)N4	LP * (6)Ag	32.19	37.78
LP(1)N4	LP * (7)Ag	10.72	13.53
**Net**		**42.91**	**51.31**
LP(1)O6	LP * (6)Ag	3.78	4.31
LP(1)O6	LP * (8)Ag	8.90	9.53
LP(2)O6	LP * (6)Ag	9.54	11.40
LP(2)O6	LP * (8)Ag	10.76	12.18
**Net**		**32.98**	**37.4**
LP(1)O5	LP * (6)Ag	3.27	4.08
LP(1)O5	LP * (9)Ag	8.59	9.94
LP(2)O5	LP * (6)Ag	3.61	4.27
LP(2)O5	LP * (9)Ag	4.64	5.40
**Net**		**20.11**	**23.7**

Bold for the net interaction energies.

**Table 7:** The occupancy and energies of the interacting NBOs incorporated in the Ag–N and Ag–O interactions.

NBO	[AgL(NO_3_)]_2_		Free Species	
	Occup.	Energy	Occup.	Energy
**B3LYP**				
LP(1)N1	1.8252	−0.4110	1.9211	−0.3478
LP(1)N1	1.8276	−0.4131	1.9211	−0.3518
LP(1)O6	1.9578	−0.6832	1.9850	−0.5378
LP(2)O6	1.8922	−0.3633	1.9087	−0.0441
LP(1)O5	1.9603	−0.7380	1.9856	−0.5423
LP(2)O5	1.8957	−0.2981	1.9155	−0.0406
LP * (6)Ag	0.2571	0.0119	0.0000	45.8839
LP * (7)Ag	0.0693	0.2325	0.0000	−0.1125
LP * (8)Ag	0.0603	0.1036	0.0000	0.5923
LP * (9)Ag	0.0494	0.0874	0.0000	3.0170
**WB97XD**				
LP(1)N1	1.8356	−0.4848	1.9245	−0.4290
LP(1)N1	1.8377	−0.4872	1.9244	−0.4331
LP(1)O6	1.9579	−0.7686	1.9853	−0.6334
LP(2)O6	1.8964	−0.4460	1.9103	−0.1259
LP(1)O5	1.9605	−0.8279	1.9859	−0.6378
LP(2)O5	1.8991	−0.3769	1.9169	−0.1227
LP * (6)Ag	0.2329	0.0808	0.0000	0.7750
LP * (7)Ag	0.0706	0.3025	0.0000	36.4326
LP * (8)Ag	0.0586	0.1721	0.0000	13.2220
LP * (9)Ag	0.0471	0.1535	0.0000	−0.0243

## Supplementary Materials

The following are available online, Figure S1. The shape index and curvedness Hirshfeld surfaces of the studied silver(I) complex; Figure S2. The full and decomposed fingerprint plots of the intermolecular contacts observed in the crystal structure of the studied silver(I) complex; Figure S3. The insignificant C…C interactions appeared as blue regions in the decomposed d_norm_ map; Figure S4. The weak Cl…H and insignificant N…H interactions appeared as fad red spots and blue regions, respectively, in the decomposed d_norm_ map; Figure S5. FTIR spectra of the ligand (L) and its silver complex; Figure S6. ^1^H-NMR spectra of the studied silver(I) complex; Table S1. The calculated natural charges using B3LYP and WB97XD methods.
